# The pandemic characteristics of 2019-nCoV: case-control study for severity and geographic locations for 2019-nCoV epidemics worldwide

**DOI:** 10.1186/s13052-020-00856-x

**Published:** 2020-07-08

**Authors:** Roberto Ronchetti, Pietro Massimiliano Bianco

**Affiliations:** 1 University “La Sapienza”, Rome, Italy; 2 Istituto Superiore Protezione Ricerca Ambiente, Rome, Italy

**Keywords:** Coronavirus, 2019-nCoV, COVID-19 outbreak, Epidemics, Pathway of spread, Severity of epidemics, Geographic location of epidemics

## Abstract

Using available official data we found 248 epidemics curves caused worldwide by the 2019-nCoV in the period December 2019–March 31st 2020. The analysis of this material allowed two main observations: 1) it is possible to describe the main geographical pathway of the diffusion of the virus in different directions. This strongly suggests a unique point of origin of the pandemics in Wuhan, China, from where it spread in many different directions. 2) of the 74 epidemics which were characterized by at least 1000 cases, 65 (90%) were located in the geographic region of the world delimitated by 52–30 degrees latitude North. Viceversa 110 (60%) of the 176 epidemics with less than 1000 cases were located outside the cited geographical world region. These results suggest considerations on the pandemic characteristics of 2019-nCoV.

The epidemic which started in December 2019 in Wuhan, China, caused by the appearance of a completely new virus, 2019-nCoV, has put humanity under a terrible, alarming threat: as the virus spread across the globe with incredible rapidity, causing a high number of epidemics frequently associated with an important mortality rate, the whole picture is confused and difficult to be described even by the Experts. To date, there has been no confirmation as to whether this terrible phenomenon will eventually slow down, completely disappear or persist in a latent form over the years to come. This situation causes in the population fear and anxiety. These feelings are the opposite to clearness and concrete programs necessary in order to obtain the right reaction and the “resilience” of the society and of single persons.

We felt that a rational approach to the issue of the “pandemic” could be achieved by means of a comprehensive analysis of the many data made available by International Agencies, Governments and local Authorities in order to concretely describe the different epidemics so far appeared in the world. We found reliable official data published before 1st April 2020 that describe 248 epidemic curves which took place worldwide.

By means of an initial preliminary analysis of this material, we have obtained two key pieces of information.

1. the geographic spreading of 2019-nCoV:

Table 1 shows that the epidemic which took place in Wuhan start in the last week of December 2019 and lasted about 10 weeks, was the initial diffusion point of the virus which then spread in a radial form in all directions showing a sequence of dates that leave no doubts on the epidemical progression of the Chinese virus. (Table 1) [1–7].

**Table 1:**
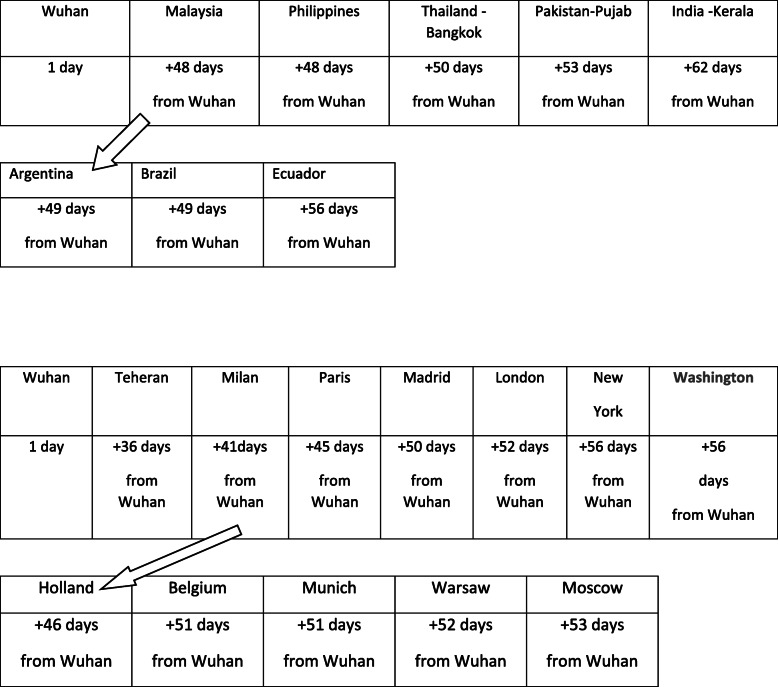
This hypothetical route of the virus from Wuhan to other locations is based on the sequences of starting days of each epidemic desumed by global and national official data. Days of interval between of the beginning of Wuhan’s epidemic and the beginning of each local epidemic

The first diffusion happened towards Northeast, with epidemics in South Korea and Japan.

The second very relevant line of diffusion took place Westward with the Teheran epidemic, which started over a month after Wuhan’s: in the space of less than a month, this second diffusion line reached Milan, Paris, Madrid, London, Ireland, New York and Washington.

A collateral arm of this line of diffusion seems to start from Paris on day 45 reaching on subsequent days, Holland, Germany, Poland and Russia.

Other diffusion line simultaneously took place towards countries located Southeast of China, South Asia and South America.

2) geographic location of the severe epidemics

After a careful analysis of the distribution of the 248 epidemics, we compiled the table shown in Table 2 which subdivides the 248 epidemics in groups based on cases number and based on the geographic area concerned (Table 2) [1–7].

**Table 2:** 90% of epidemics with over 1000 cases (panel a) took place in the area located between latitude 52 and 30 degrees North. Viceversa 62% of mild epidemics (panel D) took place outside this geographical region (data from national official sites). For details see Appendix

Epidemics 2019-nCoV	At least > 1000 cases	Less than 1000 cases	Total
Epidemics in the area 52°-30° Latitude North	65 Panel A	66 Panel C	131
In any other geographic areas	7 Panel B	110 Panel D	117
Total	72	176	248

It can be seen that the vast majority of the severe epidemics, those with over 1000 cases, are located in a very limited area of the globe, where over the last 2–3 months there have been a temperate winter meteorological conditions. Moreover if we consider the epidemics which simultaneously have more than over 1000 cases and a mortality rate above 3%,the preferential location in the area with winter climate is even more evident (data not shown).

In conclusion, these observations allow us to confirm that 2019-nCoV, starting from a single epidemic point of origin is characterized by an extremely high spreading capacity and therefore it, for sure, must be considered a virus with “pandemic” character. In terms of number of cases/epidemic (more or less than 1000 cases according to our arbitrary definition) most of the epidemics proved to be mild (176/248 = 70%). So, we conclude that most of the epidemics caused by the virus those which allow to classify the 2019-nCoV as a pandemic infective agent, are mild and for this reason these widespread infections represent the best natural form of immunization for the human beings exposed to a virus antigenically unknown.

The second main conclusion of our study deals with the remaining 30% of epidemics which were severe (> 1000 cases). The vast majority of them (65/72 = 90%) took place in a narrow geographical area included between 30°-52° latitude North. Consequently the virus activity for what concerns the severity of the epidemics appears to be heavily conditioned by “environmental factors”. This property of the virus, which in a way contradicts its pandemic nature, is not easily explicable. At first sight it could be suggested that meteorological condition modulate the virulence of the virus. It is important, however, to note that inside the 30–52° latitude North, together with the severe epidemics took place also a high number of epidemics which were not severe. Therefore this geographic location of an epidemic does not alone determine the its severity: the geographic location is almost “necessary” for an epidemic to be severe but is not “sufficient” because inside the belt 50% of the epidemics are mild. So it is evident that other environmental factors, a side of the meteorological conditions are at play. These additional factors are at the moment unknown or speculative (density of population, air pollution, etc). Our kind of study cannot contribute to clarify this item.

A question which can be finally raised is if we have, in our study, elements in order to foresee the future of the pandemic in the planet. It is relevant that those epidemics which started in January or February 2020, have, in some cases, concluded their lives: this is the cases of epidemics of Wuhan, two towns in South Korea and in Australia (Queensland). In these places the epidemic wave had a bell shape which lasted from the beginning, 5–10 weeks. We did not find, in those cases, official notices of local rebound of new cases of infection in the last several weeks after the end of the epidemic. The many other epidemic curves which started later in time have not yet reached their end but looking at the quoted examples we can optimistically believe that everywhere in the world, the virus after the end of the actual epidemic wave will become silent.

The tragic and challenging global event dealing with 2019-nCoV could affect all human behaviours: in these circumstances, where maybe 200,000 people have lost their lives, public Authorities and people accepted the employment of strict social measures and behavioural rules that have certainly widely limited the number of victims.

However, it is the very first time that political powers, international finance and humankind as a whole, have accepted to face serious economic losses in order to save human lives.

But 2019-nCoV is undeniably not the only and major factor causing avoidable deaths in the planet.

## Supplementary information

**Additional file 1.**

## Data Availability

at Prof Ronchetti’s study.
