# Supplementary material for: The pandemic characteristics of 2019-nCoV: case-control study for severity and geographic locations for 2019-nCoV epidemics worldwide
Source: Ital J Pediatr. 2020 Jul 8;46:94. doi: 10.1186/s13052-020-00856-x (PMC7341479; doi:10.1186/s13052-020-00856-x)
Supplement: Supplementary file 1 — Additional file 1. [file 13052_2020_856_MOESM1_ESM.docx]

The following list of epidemics in different places of the word have been used to compile panels of table 2.

Panel A of Table 2

Epidemics which took place at latitude 30°-52° North and with >1000 cases

| N° | Places | N° cases |
| --- | --- | --- |
| 1 | New York-USA | 213.779 |
| 2 | New Jersey-USA | 68.824 |
| 3 | Madrid-Spain | 44.783 |
| 4 | Bayern-Deuchland | 32.282 |
| 5 | Catalonia-Spain | 31.824 |
| 6 | Nordrhein Westfalen- Deuchland | 24.267 |
| 7 | Baden Württemberg- Deuchland | 24.078 |
| 8 | Michigan-USA | 22.783 |
| 9 | Switzerland | 22710 |
| 10 | Massachusetts-USA | 20.974 |
| 11 | Illinois-USA | 17887 |
| 12 | Flanders-Holland | 15384 |
| 13 | Castilla–La Mancha-Spain | 13.063 |
| 14 | Austria | 12969 |
| 15 | Milan-Italy | 12.479 |
| 16 | Istanbul-Turkey | 12231 |
| 17 | Texas-USA | 12186 |
| 18 | ÎledeFrance (Paris) | 12.108 |
| 19 | Georgia | 11859 |
| 20 | Quebec-Canada | 11677 |
| 21 | Castile and León-Spain | 11.102 |
| 22 | California-USA | 10.701 |
| 23 | Connecticut-USA | 10538 |
| 24 | Washington-USA | 10.195 |
| 25 | Brescia-Italy | 10.122 |
| 26 | Bergamo-Italy | 10.043 |
| 27 | Israel | 9404 |
| 28 | North Portugal | 8897 |
| 29 | Wallonia-Belgium | 8031 |
| 30 | London-United Kingdom | 7121 |
| 31 | Daegu-South Korea | 6.624 |
| 32 | Ontario_USA | 6237 |
| 33 | Grand Est-France | 5479 |
| 34 | Czechia | 5312 |
| 35 | Teheran-Iran | 5098 |
| 36 | Cremona-Italy | 4.489 |
| 37 | Berlin- Deuchland | 4.349 |
| 38 | Lisbon-Portugal | 3821 |
| 39 | Monza-Italy | 3.355 |
| 40 | Tirol-Austria | 3176 |
| 41 | Luxembourg | 3034 |
| 42 | Roma-Italy | 3.026 |
| 43 | Pavia-Italy | 2.889 |
| 44 | Brussels-Belgium | 2842 |
| 45 | Lodi-Italy | 2.376 |
| 46 | Lower Austria | 2213 |
| 47 | Center-Austria | 2197 |
| 48 | Upper Austria | 2109 |
| 49 | Auvergne -France | 2.093 |
| 50 | Pesaro-Italy | 2044 |
| 51 | Esfahan-Iran | 1979 |
| 52 | Vienna-Austria | 1963 |
| 53 | Provence-France | 1.924 |
| 54 | Brandenburg- Deuchland | 1920 |
| 55 | Greece | 1884 |
| 56 | Hauts de France | 1.753 |
| 57 | Suceava-Romania | 1661 |
| 58 | Styria-Austria | 1499 |
| 59 | Ancona-Italy | 1481 |
| 60 | Hart voor Brabant-Holland | 1344 |
| 61 | Gyeongbuk-South Korea | 1298 |
| 62 | Morocco | 1275 |
| 63 | Gilan-Iran | 1191 |
| 64 | Salzburg-Austria | 1154 |
| 65 | Slovenia | 1091 |

Panel B of Table 2

Epidemics which took place outside 30°-52 North and with > 1000 cases

|  | Places | Cases |
| --- | --- | --- |
| 1 | Ecuador | 2758 |
| 2 | São Paulo - Brazil | 2339 |
| 3 | South Wales - Australia | 2182 |
| 4 | Saudi Arabia | 1720 |
| 5 | Santiago - Chile | 1521 |
| 6 | Peru | 1323 |
| 7 | Manila - Philippines | 1186 |

Panel C of Table 2

Epidemics which took place at latitude 30°-52° North and with < 1000 cases

|  | Places | Cases |
| --- | --- | --- |
| 1 | Hungary | 980 |
| 2 | Azerbaijan | 926 |
| 3 | Armenia | 921 |
| 4 | Bucharest– Romania | 870 |
| 5 | Punjab - Pakistan | 845 |
| 6 | Saxony-Deuchland | 750 |
| 7 | Kazakhstan | 727 |
| 8 | Utrecht- Netherlands | 716 |
| 9 | Slovakia | 682 |
| 10 | North Macedonia | 617 |
| 11 | Brabant Zuidoost- Netherlands | 599 |
| 12 | Bulgaria | 593 |
| 13 | Lebanon | 575 |
| 14 | Rotterdam-Rijnmond- Netherlands | 569 |
| 15 | Andorra | 566 |
| 16 | Uzbekistan | 555 |
| 17 | Blida- Algeria | 529 |
| 18 | Cyprus | 526 |
| 19 | Amsterdam- Netherlands | 500 |
| 20 | Kanto- Japan | 445 |
| 21 | Frosinone - Italy | 441 |
| 22 | Silesia - Poland | 431 |
| 23 | Albania | 409 |
| 24 | Lower Silesia -Poland | 400 |
| 25 | Latina - Italy | 377 |
| 26 | Jordan | 358 |
| 27 | Belgrade- Serbia | 352 |
| 28 | Blida- Algeria | 342 |
| 29 | Kansai- Japan | 339 |
| 30 | Algiers- Algeria | 310 |
| 31 | San Marino-Italy | 308 |
| 32 | Malta | 299 |
| 33 | Kyrgyzstan | 280 |
| 34 | Rieti-Italy | 278 |
| 35 | Khyber Pakhtunkhwa- Pakistan | 276 |
| 36 | Palestinian | 263 |
| 37 | Baja Calif.- Mexico | 258 |
| 38 | Montenegro | 248 |
| 39 | Kosovo | 224 |
| 40 | Chubu- Japan | 218 |
| 41 | Viterbo-Italy | 216 |
| 42 | Georgia | 214 |
| 43 | Herat Province- Afghanistan | 204 |
| 44 | Kiev- Ukraine | 180 |
| 45 | Baghdad - Iraq | 180 |
| 46 | Banja Luka- Bosnia | 171 |
| 47 | Chernivtsi Oblast- Ukraine | 171 |
| 48 | Jersey | 170 |
| 49 | Chişinău - Moldova | 170 |
| 50 | Guernsey | 166 |
| 51 | Split-Croazia | 133 |
| 52 | Najaf -Iraq | 121 |
| 53 | Ternopil Oblast- Ukraine | 114 |
| 54 | Gibraltar | 113 |
| 55 | Tunis- Tunisia | 104 |
| 56 | Oran- Algeria | 104 |
| 57 | Liechtenstein | 79 |
| 58 | Istria-Croazia | 71 |
| 59 | Ariana- Tunisia | 57 |
| 60 | Monaco | 54 |
| 61 | Ştefan Vodă - Moldova | 50 |
| 62 | Soroca - Moldova | 41 |
| 63 | Bermuda | 39 |
| 64 | Saint Martin | 31 |
| 65 | Laktaši-Bosnia | 19 |
| 66 | Mongolia | 16 |

Panel D of Table 2

Epidemics which took place outside 30°-52 North and with < 1000 cases

| N° | Places | Cases |
| --- | --- | --- |
| 1 | Victoria- Australia | 968 |
| 2 | Singapore | 926 |
| 3 | Jakarta- Indonesia | 897 |
| 4 | Helsinki - Finland | 836 |
| 5 | United Arab Emirates | 814 |
| 6 | Masovia - Poland | 808 |
| 7 | Selangor - Malaysia | 800 |
| 8 | Viken- Norwey | 799 |
| 9 | New Zealand | 797 |
| 10 | Queensland- Australia | 781 |
| 11 | Sindh- Pakistan | 743 |
| 12 | Oslo- Norwey | 720 |
| 13 | Rio de Janeiro- Brazil | 708 |
| 14 | Bangkok - Thailand | 647 |
| 15 | Bahrain | 567 |
| 16 | Panama | 558 |
| 17 | Qatar | 549 |
| 18 | Estonia | 538 |
| 19 | Dominican Republic | 488 |
| 20 | Kuala Lumpur - Malaysia | 488 |
| 21 | Cuba | 457 |
| 22 | Latvia | 446 |
| 23 | Silesia - Poland | 431 |
| 24 | Uruguay | 424 |
| 25 | Johor- Malaysia | 395 |
| 26 | Buenos Aires- Argentina | 387 |
| 27 | Lithuania | 358 |
| 28 | Kuwait | 342 |
| 29 | Distrito Federal-Brazil | 332 |
| 30 | Puerto Rico | 316 |
| 31 | Costa Rica | 314 |
| 32 | Honduras | 312 |
| 33 | Mexico City-Mexico | 296 |
| 34 | Burkina Faso | 282 |
| 35 | Maharashtra-India | 254 |
| 36 | Vietnam | 227 |
| 37 | West Java- Indonesia | 223 |
| 38 | Kerala-India | 216 |
| 39 | Ghana | 204 |
| 40 | Senegal | 195 |
| 41 | Cote d’Ivoire | 190 |
| 42 | Kiev- Ukraine | 180 |
| 43 | Niger | 174 |
| 44 | Nigeria | 174 |
| 45 | Southern Thailand-Thailand | 174 |
| 46 | Faroe Islands | 169 |
| 47 | Belarus | 163 |
| 48 | State of Mexico- Mexico | 157 |
| 49 | Sri Lanka | 150 |
| 50 | Réunion | 145 |
| 51 | Cameroon | 139 |
| 52 | Congo | 134 |
| 53 | Dem. Rep. of the Congo | 134 |
| 54 | Brunei Darussalam | 129 |
| 55 | Mayotte | 128 |
| 56 | Tamil Nadu-India | 119 |
| 57 | Paraguay | 119 |
| 58 | Guadeloupe | 114 |
| 59 | Delhi-India | 112 |
| 60 | Cambodia | 110 |
| 61 | Trinidad and Tobago | 107 |
| 62 | Jalisco-Mexico | 99 |
| 63 | Pirkanmaa-Finland | 97 |
| 64 | El Salvador | 93 |
| 65 | Martinique | 93 |
| 66 | Guatemala | 87 |
| 67 | Guam | 82 |
| 68 | Kenya | 81 |
| 69 | Mauritius | 81 |
| 70 | Rwanda | 75 |
| 71 | Barbados | 63 |
| 72 | Jamaica | 63 |
| 73 | Bangladesh | 57 |
| 74 | Miranda-Venezuela | 56 |
| 75 | Aruba | 55 |
| 76 | French Guiana | 55 |
| 77 | Guinea | 52 |
| 78 | Djibouti | 41 |
| 79 | Oman | 39 |
| 80 | Zambia | 39 |
| 81 | French Polynesia | 37 |
| 82 | Bahamas | 36 |
| 83 | Guyana | 33 |
| 84 | United States Virgin Islands | 33 |
| 85 | Cayman Islands | 28 |
| 86 | Ethiopia | 26 |
| 87 | Capital District-Venezuela | 25 |
| 88 | Togo | 24 |
| 89 | Cochabamba-Bolivia | 21 |
| 90 | Tanzania | 20 |
| 91 | Aragua-Venezuela | 20 |
| 92 | Maldives | 19 |
| 93 | Gabon | 18 |
| 94 | New Caledonia | 18 |
| 95 | Saint Martin | 18 |
| 96 | La Paz-Bolivia | 16 |
| 97 | Equatorial Guinea | 15 |
| 989 | Benin | 13 |
| 99 | Saint Lucia | 13 |
| 100 | Greenland | 10 |
| 101 | Seychelles | 10 |
| 102 | Eswatini | 9 |
| 103 | Central African Republic | 8 |
| 104 | Suriname | 8 |
| 105 | Chad | 7 |
| 106 | Curaçao | 7 |
| 107 | British Virgin islands | 7 |
| 108 | Sudan | 7 |
| 109 | Liberia | 6 |
| 110 | Nepal | 6 |
